# Energy Landscape Analysis of Membrane Proteins Using NMR-Based Hybrid Restraint Potentials

**DOI:** 10.1021/acs.jctc.5c02070

**Published:** 2026-03-12

**Authors:** Diksha Dewan, Yifei Wang, Alfonso De Simone, David J. Wales

**Affiliations:** † Yusuf Hamied Department of Chemistry, 2152 University of Cambridge, Lensfield Road, Cambridge CB2 1EW, U.K.; ‡ Department of Pharmacy, 9307 University of Naples Federico II, Naples 80131, Italy

## Abstract

Most biomolecular simulations depend on the quality of empirical force fields, and the use of hybrid restraint potentials has emerged as a promising approach. In this contribution, we extend the application of hybrid potentials to membrane proteins by developing optimized restraints derived from experimentally determined NMR data. NMR chemical shift, chemical shift anisotropy, dipolar coupling, and NOE distance information are combined with appropriately weighted empirical force fields to study two transmembrane systems, namely sarcolipin and phospholamban. To remedy the problems of rare events and broken ergodicity, the energy landscape framework, including basin-hopping global optimization and discrete path sampling, is employed for exploring the underlying energy landscapes. Much of the appeal of the hybrid potential approach is the ability to study membrane proteins in the absence of conventional explicit or implicit solvent and lipid molecules, thereby simplifying the sampling of complex biomolecular conformational spaces. Our results suggest that the hybridization of NMR constraints as penalty energies with empirical force fields improves global optimization and energy landscape analysis by excluding experimentally incompatible structures.

## Graphical abstract

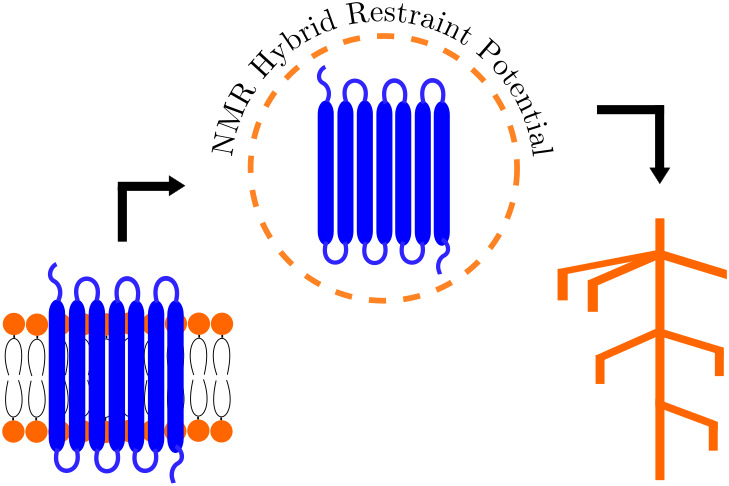

## 1 Introduction

Deciphering the intricate relationship between the structural and dynamical properties of proteins and their biological significance constitutes the central pillar of structural biology, and underpins the pharmaceutical design industry. Major experimental techniques for protein structure determination include X-ray crystallography, cryo-electron microscopy and nuclear magnetic resonance (NMR) spectroscopy.^1^ NMR is particularly sensitive to variations in chemical environments and has the ability to shed light on both the structure and dynamics on various time scales, making it a powerful tool for biomolecular analysis.^2^ Furthermore, solid-state NMR (ssNMR) enables the characterization of biomolecules within their native environments, a capability that is particularly significant for membrane proteins, as their structural and functional features are partly determined by lipid interactions.^3^ However, as of June 2025, the overall contribution of NMR to the protein structure database in RCSB PDB accounts for just 6.2% of the total entries.^4,5^ Therefore, developing tools to better exploit NMR experimental information is an important research challenge.

Standard NMR methods for biomolecular studies typically consist of four key stages: (1) sample preparation including isotopic labeling; (2) collection of NMR data, including chemical shift resonances, dipolar coupling values, nuclear Overhauser effect (NOE) derived distances, etc.; (3) conversion of NMR data into structural models; (4) assessment of the quality of the resulting configurations.^2,6,7^ At the core of this workflow is the use of NMR data to infer the structural and orientational characteristics of a protein native state. Several studies have dealt with this challenge in diverse ways. One approach is “molecular fragment replacement”,^8^ where structural fragments are extracted based on sequence homology information, and then reassembled into full conformations. CS-ROSETTA^9^ is a computational method that involves extraction of compatible polypeptide fragments from databases based on experimentally determined chemical shift values and the amino acid sequence. This procedure is followed by molecular fragment replacement to build the target biomolecule. Such techniques, however, do not support detailed conformational space sampling.^10,11^

A promising approach to extract structural information from raw NMR data is to use restraint potentials. This approach involves calculation of NMR observables using either physical models or fitted artificial neural networks, and comparing with the respective experimentally available values to guide the conformational sampling. For example, various tools been developed to process chemical shift data and relate those results to structural features. SHIFTX^12^ is one of the earliest computer programs aimed at calculating chemical shift values, and it makes use of both empirical chemical shift hypersurfaces and semiempirical relationships. CamShift,^13^ developed by Vendruscolo and coworkers, makes use of a polynomial expansion-based prediction procedure, where atomic chemical shifts are approximated using interatomic distances. Recently, some machine learning based approaches have also been introduced for this purpose. For instance, SPARTA+^14^ is based on artificial neural networks and considers subtle details like ring effects for aromatic systems, hydrogen-bonding, and electric fields.

Membrane proteins (MPs) form an ideal testing ground for such hybrid restraint potentials. MPs play crucial roles in various essential biological processes, including communication between extracellular and intracellular regions, signal transduction, energy conversion, nutrient and waste transport, and electrical current conduction.^15–17^ Their significance is underscored by the fact that they constitute about one-third of all proteins encoded by the genomes of most organisms, and consequently, are a major target for biomedical research, with significant potential for therapeutic applications.^18,19^ This approach has been exemplified by the orientationally constrained replica-averaged molecular dynamics (MD) runs carried out by De Simone et al.,^20^ based on chemical shift anisotropy and dipolar coupling observables, which were applied to the pentameric state of the transmembrane (TM) protein phospholamban. This contribution included studies in the presence of explicit DOPC:DOPE lipid bilayer. However, despite their importance, computational studies on MPs are normally limited, owing to the bulk lipid membrane environments, which is a major computational bottleneck, especially for larger MPs. Furthermore, since MPs are inherently dynamic molecules that exhibit complex conformational changes, sampling their conformational space involves rare events and broken ergodicity problems.

Our work addresses these issues by incorporating the hybrid restraint potential approach within the energy landscape framework,^21^ which has proved effective in three key areas: structure prediction, enhanced thermodynamic sampling, and characterization of rare event dynamics.^22,23^ We employ the energy landscape framework to accomplish the central objective of this project, which is to address the problem of biomolecular structure prediction where analogous structural information is lacking and where additional theory and computational tools are needed to better exploit the experimentally available data. We develop optimized NMR-based hybrid restraint potential energy functions as a novel approach to study membrane proteins, which provides a much less complicated, yet realistic, description of the membrane and solvent compared to the standard explicit or implicit environments. We consider chemical shift, chemical shift anisotropy, dipolar coupling, and NOE measurements, coupled with appropriately weighted force field potentials, including AMBER ff19SB and CHARMM36m, to enhance structure prediction and energy landscape analysis using basin-hopping global optimization^24^ and discrete path sampling.^25^ We test this method on two integral membrane protein systems, namely, sarcolipin (SLN) and phospholamban (PLN). The structure and orientation biasing capabilities of different NMR hybrid potentials are explored. We also present an analysis of the variation in underlying potential energy landscapes for the folding pathway of SLN with addition of NMR constraints. We further examine the capabilities of the hybrid potential setup to study the conformational state switching pathway for PLN. Overall, our results highlight the effectiveness of combining multiple NMR constraints with empirical biomolecular force fields as hybrid potentials to improve landscape analysis methods for membrane proteins. Furthermore, use of NMR observables can be effective in describing the local chemical environments for MPs without making use of implicit/explicit lipid and solvent molecules.

## 2 Methods

### 2.1 Hybrid Potential

We employ the following hybrid potential based on NMR observables:

1 $$E_{\mathrm{Total}}=E_{\mathrm{FF}}+\underset{\alpha }{\sum }w_{\alpha }E_{\mathrm{res}}^{\alpha }$$

*E*_Total_, *E*_FF_, and *E*_res_ refer to the total potential energy, atomistic force field energy, and NMR penalty energy, respectively. α represents different NMR observable types, and *w*_α_ corresponds to the weights associated with each type introduced as restraint. These weights are system dependent and allow precise control over the degree of bias introduced during sampling. The weight *w*_α_ is dimensionless, while *E*_res_ has units of kcal mol^–1^. At each simulation step, the penalty energy is calculated and then differentiated analytically with respect to atomic coordinates, generating a force that introduces the required experimental data.

Incorporating NMR constraints helps correct systematic errors in the underlying force fields, e.g., overstabilization of certain secondary structural elements, and enhances the efficiency of conformational space sampling by excluding regions that are experimentally incompatible. Furthermore, another significant advantage is the representation of near-native environments for membrane proteins, without relying on the presence of implicit or explicit lipid and solvent molecules or a coarse-grained description. This approach offers an efficient alternative to investigate membrane proteins. In the current study, we implemented a hybrid potential corresponding to dipolar coupling and chemical shift anisotropy in the GMIN^26^ and OPTIM^27^ programs for basin-hopping global optimization and discrete path sampling, respectively.

### 2.2 Chemical Shift

Resonance frequencies of nuclei are a function of both the external magnetic field and the surrounding chemical environments. The variation in resonance frequencies, arising from diverse local molecular environments, is quantified using the isotropic chemical shift (CS). For a magnetically active nucleus with resonance frequency ν_*i*_, the chemical shift δ_*i*_ is measured in parts per million (ppm), and is defined based on a reference nucleus of resonance frequency ν_ref_ as^28^

2 $$\delta_{i}=10^{6}\left(\nu_{i}-\nu_{\mathrm{ref}}\right)/\nu_{\mathrm{ref}}$$

This NMR observable changes distinctly with molecular geometry, electronegativity of different atoms, bond types, and bond hybridization, etc.^29^ Here, we exploit the capabilities of CS data in predicting secondary structure elements in proteins, in combination with orientational information from other NMR observables.

To achieve efficient mapping between CS and structure, we calculated CS at different steps in our simulations using the artificial neural network NapShift.^30^ This model is fitted to predict CS values for a specific set of magnetically active nuclei from a given protein structure and amino acid sequence. NapShift consists of six independent, feed-forward, fully connected single layer neural networks for six CS types ^15^N, ^13^C, ^13^C_α_, ^13^C_β_, ^1^H, and ^1^H_α_. The output is a continuous real number for each of the six atoms, which is the secondary CS. Each type of atom in every amino acid has a characteristic baseline CS value, i.e, random coil CS, which is an average of all conformations possible for an amino acid in the absence of any secondary structure element. The CS value dependent on the secondary structure of a protein is thus called the secondary CS △δ, and is related to the random coil δ_rc_ and experimentally observed CS δ_obs_ as

3 $$△\delta =\delta_{\mathrm{obs}}-\delta_{\mathrm{rc}}$$

We used the Fortran implementation of this model in conjunction with basin-hopping and discrete path sampling, and converted the differences between back-calculated and experimental CS to a penalty energy that is differentiable with respect to atomic coordinates via a flat-bottom constraint potential, with a harmonic constant *k*_CS_. This formulation introduces experimental constraints and accounts for errors associated with the NapShift artificial neural network and experimental measurements, and scales the CS restraint energy (*V*_CS_) as

4 $$V_{\mathrm{CS}}=k_{\mathrm{CS}}\overset{N_{\mathrm{res}}}{\underset{i}{\sum }}\overset{6}{\underset{j}{\sum }}{(\delta_{ij}^{\exp t}-\delta_{ij}^{\mathrm{calc}})}^{2}$$

*N*_res_, $\delta_{ij}^{\mathrm{expt}}$ , and $\delta_{ij}^{\mathrm{calc}}$ denote the number of residues, experimental secondary CS, and NapShift back-calculated secondary CS, respectively. Qi et al. offers a thorough description of NapShift’s architecture.^30^ A tolerance parameter (ϵ) is taken as input from the user to adjust the size of the flat-bottom region as

5 $$\delta_{ij}^{\mathrm{calc}}-\delta_{ij}^{\mathrm{expt}}=\{\begin{array}{ll} \delta_{ij}^{\mathrm{calc}}-\delta_{ij}^{\mathrm{expt}}, & \mathrm{if}\;\delta_{ij}^{\mathrm{calc}}>\delta_{ij}^{\mathrm{upp}},\\ 0, & \mathrm{if}\;\delta_{ij}^{\mathrm{low}}\leq \delta_{ij}^{\mathrm{calc}}\leq \delta_{ij}^{\mathrm{upp}},\\ \delta_{ij}^{\mathrm{calc}}-\delta_{ij}^{\mathrm{expt}}, & \mathrm{if}\;\delta_{ij}^{\mathrm{calc}}<\delta_{ij}^{\mathrm{low}}, \end{array}$$

where

6 $$\begin{array}{ll} & \delta_{ij}^{\mathrm{upp}}=\delta_{ij}^{\mathrm{expt}}+\epsilon \left({\mathrm{Error}}_{j}\right)\\ & \delta_{ij}^{\mathrm{low}}=\delta_{ij}^{\mathrm{expt}}-\epsilon \left({\mathrm{Error}}_{j}\right) \end{array}$$

are the upper and lower bounds for the flat-bottom region. Error_*j*_ denotes the root-mean-square deviation of the NapShift predicted value for atom type *j*, with respect to the training set, in ppm. The overall contribution from the CS penalty energy *V*_CS_ and force field energy *V*_FF_ in the total hybrid energy *V*_Total_ was tuned using a mixing parameter (or weight), α_CS_, as

7 $$V_{\mathrm{Total}}=V_{\mathrm{FF}}+\alpha_{\mathrm{CS}}V_{\mathrm{CS}}$$

In the present work, we employ NapShift for the study of membrane proteins. Previously, Napshift has already been explicitly validated on non-native and partially folded protein states, including conformational ensembles that deviate substantially from the native structure. In particular, we found that NapShift accurately reproduced experimental chemical shifts across heterogeneous ensembles containing partially folded and dynamically interconverting conformations, demonstrating applicability beyond native-state structures.^31^ It should also be noted that the CS restraints primarily report on local backbone and side-chain environments, which are captured by the structural descriptors employed in NapShift and are not intrinsically dependent on whether the protein is embedded in a membrane or in solution. As a result, the predictor remains applicable to membrane-associated systems, including conformationally heterogeneous and non-native states, provided that the underlying local geometry is sampled. Furthermore, to account for potential systematic errors from NapShift, we have applied the CS restraints in combination with the empirical force field and, where appropriate, with complementary NMR observables, which together reduce the impact of any residual bias from a single data type. These NMR restraints are explained below.

### 2.3 Dipolar Coupling

Dipolar coupling is a through-space effect that measures the interaction between one nuclear spin and the magnetic field produced by another, in the presence of an external magnetic field. For two spins *I* and *S* and a magnetic field oriented along the *z*-axis (by convention), the strength of the dipolar interaction between the two spins is defined by the dipolar coupling constant, denoted by DC:^32^

8 $$\mathrm{DC}=-\frac{\mu_{0}\gamma_{I}\gamma_{S}h}{16\pi^{3}r_{IS}^{3}}\left(3\cos^{2}\theta -1\right)$$

where μ_0_ is the magnetic permeability of free space (4π × 10^–7^ N A^–2^), γ denotes the gyromagnetic ratio of a nucleus, *h* is Planck’s constant, θ is the angle between the internuclear vector and the magnetic field, and *r*_*IS*_ is the distance between the nuclei. For an ^15^N and ^1^H pair, with γ_N_ and γ_H_ as −2.71171 × 10^7^ and 26.75105 × 10^7^ rad T^–1^s^–1^, respectively, and internuclear distance 1.32 Å, we obtain the following expression for dipolar coupling in a ^15^N–^1^H bond (DC_NH_):

9 $$DC_{\mathrm{NH}}=\frac{1}{2}\xi_{\mathrm{DC}}\left(3\cos^{2}\theta -1\right)$$

where ξ_DC_ is 10.52 kHz. This structural model has previously been used by De Simone et al., for studies on pentameric phospholamban using replica-averaged restrained MD simulations.^20,33^ Eq 9 suggests that maximum coupling is observed when the internuclear vector between the two nuclei is parallel/antiparallel to the external field. For any other orientation of the vector, the observed DC value is smaller. Since DC is an orientation-dependent interaction, it averages to zero in isotropic liquids, due to the random molecular tumbling motion. However, in oriented ssNMR experiments where the sample is embedded in alignment media such as bicelles, polyacrylamide gels etc., DC provides a wealth of orientational information.^34^ This feature underpins its utility in the three-dimensional structure prediction of proteins, along with other structural restraints, and in the combination of structural and orientational NMR data to study the structure and conformational dynamics of TMs.

We designed the GMIN and OPTIM implementation of the DC constraint using the following penalty energy:

10 $$V_{\mathrm{DC}}=k_{\mathrm{DC}}\overset{N_{\mathrm{res}}}{\underset{i}{\sum }}\overset{atom\;types}{\underset{j}{\sum }}{(DC_{ij}^{\exp t}-DC_{ij}^{\mathrm{calc}})}^{2}$$

where *k*_DC_ scales the DC restraint potential. Similar to the CS implementation, in eq 5, *V*_DC_ consists of a flat-bottom harmonic potential, where the size of the flat-bottom region is adjustable. Additionally, the relative weight of force field energy and DC penalty energy is adjusted using a mixing parameter, α_DC_, as

11 $$V_{\mathrm{Total}}=V_{\mathrm{FF}}+\alpha_{\mathrm{DC}}V_{\mathrm{DC}}$$

### 2.4 Chemical Shift Anisotropy

Another ssNMR derived orientational observable is chemical shift anisotropy (CSA). Since the electronic distribution around the nucleus lacks spherical symmetry, the CS varies with the orientation of the external field. This orientational dependence of CS is captured by CSA.^28^ In this study, we employ the CSA structural model described by De Simone et al.^33^ in the study of membrane proteins. The amide nitrogen is represented by a 3 × 3 tensor with principal elements δ_11_, δ_22_, and δ_33_, in ppm. The overall CSA of the amide nitrogen under investigation is calculated as

12 δN15=δ11×sin2(α−17)×sin2(β)+δ22×cos2(β)+δ33×cos2(α−17)×sin2(β)

Here, δ_11_, δ_22_, and δ_33_ denote the experimental components of the ^15^N amide CS tensor in the principal axis frame (PAF), while α and β represent the Euler angles (in degrees) used for the corresponding frame transformation. This model uses fixed experimentally determined values for the three components, given as δ_11_ = 640, δ_22_ = 760, δ_33_ = 2169 for nonglycine residues and δ_11_ = 465, δ_22_ = 663, δ_33_ = 2116 for the glycine residue. Fixing these values, however, could be a minor source of systematic error in further calculations.^35–37^

The GMIN and OPTIM implementation of the CSA constraint has been developed similarly to CS and DC, with a flat bottom harmonic restraint potential, as a function of the difference between experimentally derived and calculated CSA value for the amide nitrogens. The contribution is also adjusted in a similar fashion, using α_CSA_, as

13 $$V_{\mathrm{Total}}=V_{\mathrm{FF}}+\alpha_{\mathrm{CSA}}V_{\mathrm{CSA}}$$

### 2.5 Distance Constraints

The random molecular tumbling in liquids modulates dipolar interactions between nuclear spins, which further leads to a transfer of magnetization from one spin to another. This interaction is the basis of the Nuclear Overhauser Effect (NOE).^2,38^ NOE distances are conventionally noted between nearby hydrogen atoms (less than 5–6 Å) for protein structure determination. Depending on the distance between the two nuclei, these distances can be classified into intraresidue, sequential, medium-range, and long-range. The most important are the long-range NOEs, where the hydrogen atoms under consideration belong to residues separated by at least four amino acids, thus interrogating sequentially distant but spatially close regions of the protein. Experimentally, the intensity of the NOE between a pair of nuclei is quantified by the volume of the respective cross-peak in a NOESY spectrum, which is a function of the internuclear distance, given as^2,39^

14 $$V=\left\langle r^{-6}\right\rangle f\left(\tau_{c}\right)$$

where *r* denotes the separation between the two interacting hydrogen nuclei, and *f*(τ_c_) accounts for the influence of overall molecular tumbling and internal protein motions during the magnetization transfer process. NOE-derived distances are obtained from solution-state NMR and therefore represent ensemble-averaged quantities, typically defined by both upper and lower distance bounds.^2^

We incorporate the experimental NOE distance values obtained from solution NMR studies into the hybrid potential setup using a similar flat bottom harmonic potential, as for other NMR observables:

15 $$\Delta {\mathrm{Dist}}_{i}=\{\begin{array}{ll} {\mathrm{Dist}}_{i}^{\mathrm{calc}}-{\mathrm{Dist}}_{i}^{\mathrm{expt}}, & \mathrm{if}\;{\mathrm{Dist}}_{i}^{\mathrm{calc}}>{\mathrm{Dist}}_{i}^{\mathrm{upp}},\\ 0, & \mathrm{if}\;{\mathrm{Dist}}_{i}^{\mathrm{low}}\leq {\mathrm{Dist}}_{i}^{\mathrm{calc}}\leq {\mathrm{Dist}}_{i}^{\mathrm{upp}},\\ {\mathrm{Dist}}_{i}^{\mathrm{calc}}-{\mathrm{Dist}}_{i}^{\mathrm{expt}}, & \mathrm{if}\;{\mathrm{Dist}}_{i}^{\mathrm{calc}}<{\mathrm{Dist}}_{i}^{\mathrm{low}}, \end{array}$$

where

16 $$\begin{array}{ll} & {\mathrm{Dist}}_{i}^{\mathrm{upp}}={\mathrm{Dist}}_{i}^{\mathrm{expt}}+\delta \\ & {\mathrm{Dist}}_{i}^{\mathrm{low}}={\mathrm{Dist}}_{i}^{\mathrm{expt}}-\delta \end{array}$$

δ is the error tolerance and the flat-bottom size of the constraint potential and has been set to 1.0 Å in the present study. The constraint potential and the corresponding hybrid potential energy are

17 $$V_{\mathrm{Dist}}=\underset{i}{\sum }{(\Delta {\mathrm{Dist}}_{i})}^{2}$$

18 $$V_{\mathrm{Total}}=V_{\mathrm{FF}}+\alpha_{\mathrm{Dist}}V_{\mathrm{Dist}}$$

### 2.6 Energy Landscape Exploration

We explored the underlying potential energy surfaces using basin-hopping (BH)^24^ global optimization as implemented in the GMIN program.^26^ In this method, energy minimization at any point in the configurational space leads to a transformed energy surface, consisting of “basins of attraction”, where each basin consists of the configurations that lead to a particular local minimum.^21,24^ Group rotation moves were employed for stepping between local minima, where backbone and side chain dihedral angles were selected at random followed by a random angular perturbation.^40^ This perturbation was followed by minimization and then a Metropolis accept/reject criterion for the energies of the minima.^41^ All local minimizations were carried out by the limited-memory Broyden-Fletcher-Goldfarb-Shanno (L-BFGS) algorithm, an extension of the BFGS algorithm that retains a fixed number of gradients, making this procedure suitable for large scale problems.^42,43^ Metropolis temperature and step size were varied to adjust the step-taking. An acceptance ratio of approximately 30% was maintained for BH exploration, and an RMS convergence criterion for L-BFGS minimization was fixed at 10^–3^ kcal mol^–1^Å^–1^ for all BH quenches with the lowest minima further refined to an RMS gradient tolerance of 10^–6^ kcal mol^–1^Å^–1^ at the end of the run.

Further investigation of the underlying energy landscapes for NMR hybrid potentials was performed using discrete path sampling (DPS),^25,44^ where geometry optimization techniques are used to obtain databases of stationary points. Discrete paths are sequences of interconnected local minima and intervening transition states.^25,44^ Transition state candidates between a pair of selected minima were obtained using the doubly nudged^45^ elastic-band^46,47^ (DNEB) method and further subjected to tight convergence by hybrid eigenvector-following (HEF).^48^ In addition, approximate steepest-descent paths, calculated with a modified version of the L-BFGS algorithm,^42,43^ were employed to define the two local minima directly connected to each transition state. For a distinct minima pair, a fully connected path may not be achievable in a single connection attempt. We therefore employed an iterative missing-connection procedure based on Dijkstra’s shortest path,^49^ to make more connection attempt cycles, until a fully connected pathway was obtained.^50^ The DNEB and HEF methods are implemented in OPTIM,^27^ our program for transition state and pathway calculations, and the DPS scheme is implemented in PATHSAMPLE,^51^ a driver for OPTIM to initiate and organize multiple OPTIM jobs for constructing long pathways in parallel.

In the present work, we explore the folding pathways for the conversion of the fully extended structure to the folded conformation for SLN and the R- to T-state conformational change for PLN, using GMIN and OPTIM interfaced with AMBER ff19SB and CHARMM36m force fields and the hybrid potential setup. The landscapes were visualized using disconnectivity graphs^52,53^ using the disconnectionDPS program.^54^ In a disconnectivity graph, each vertical branch represents a local minimum, with the end point indicating the corresponding energy value on the vertical axis. At a given energy threshold, minima that can interconvert without surpassing this threshold are grouped into superbasins. The horizontal axis represents the overall organization of the landscape in the form of superbasins. If two minima in the kinetic transition network are connected by multiple transition states, only the transition state with the lowest energy affects the disconnectivity representation.^21,52^

### 2.7 Computation of Structural Order Parameters

We quantified the secondary structure of the proteins by calculating the number of native backbone hydrogen bonds formed within the reference helical regions, specifically between the backbone N–H group of the *i* ^th^ residue and the carbonyl oxygen of the *i*+4^th^ residue, denoted by *N*_b_. We used the COOR HBOND facility of the CHARMM^55^ program for this purpose. For SLN, the maximum value of *N*_b_ was 16, which reflects the backbone hydrogen bonds stabilizing the TM helix spanning residues 7–26 in the reference native structure, as shown in Figure 1. *N*_b_ ranged from 0 to 16, and coloring the disconnectivity graphs using this order parameter allowed a direct analysis of funnels corresponding to different conformations without any loss of structural information. In addition, the root-mean-square deviation (RMSD) and helix tilt angles relative to the experimental structures were employed as complementary order parameters.

**Figure 1:**
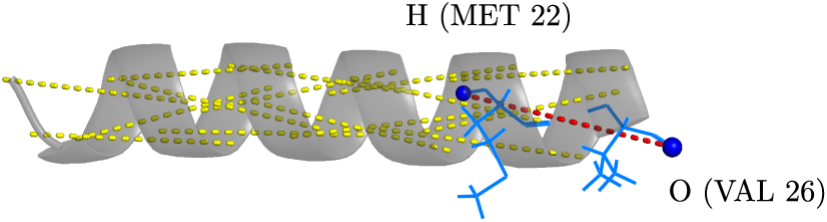
TM region of the reference SLN structure where the α-helix contains 16 backbone hydrogen bonds (depicted in yellow) formed between the backbone N–H group of the *i* ^th^ residue and the carbonyl oxygen of the *i*+4^th^ residue. For illustration, the pair MET 22 and VAL 26 is shown in blue with the corresponding hydrogen bond in red.

## 3 Results

### 3.1 Transmembrane Protein System 1: Sarcolipin

The first system investigated in this work is sarcolipin (SLN), a 31-residue TM protein with sequence MGINTRELFLNFTIVLITVILMWLLVRSYQY, expressed in the skeletal and cardiac muscles to different extents in the human body.^56^ Functionally, SLN plays an important role in the regulation of the sarco/endoplasmic reticulum Ca^2+^-ATPase (SERCA) pump for muscular relaxation and thermogenesis.^56–58^ The protein has four subdomains: an unstructured N-terminus ranging from residues 1 to 6; a dynamic helix composed of residues 7 to 14 and a more rigid helix from residues 15 to 26; followed by a short semiflexible C terminus from residues 27 to 31.^59^ Additionally, this TM protein adopts a characteristic orientation relative to the membrane normal, referred to as the tilt angle. MD simulations performed by Shi et al.^60^ in explicit dioleoylphosphocholine (DOPC) lipid membranes reported an average tilt angle of 28 ± 6°. Subsequent studies carried out by De Simone et al.^33^ on SLN in a DOPC environment, using single-replica restrained MD simulations with ssNMR restraints based on DC and CSA data, suggested a tilt angle of 23°.

We performed energy landscape analysis on SLN without any explicit/implicit lipid or solvent molecules, using experimentally available CS and DC data in hybrid restraint potentials, to examine the capability of NMR constraints in defining the local environment consisting of lipid and water molecules. For all studies carried out on this TM protein, the semiflexible amino (N-terminal) and carboxyl (C-terminal) ends were capped with acetyl (CH_3_CO–, referred to as ACE) and *N*-methylamide (−NHCH_3_, referred to as NME) groups, respectively. Introducing capping on terminal groups was deemed necessary because in the absence of stabilizing hydrophilic lipid heads and polar water molecules around the protein, the strong electrostatic interactions between the charged terminal groups induce bending into SLN helix, thus producing high penalty energies from the constraints. This interaction between the termini is rather artificial in the absence of a stabilizing environment, and lowers the efficiency of global optimization. Figure 2 shows the lowest-energy structure obtained from a 20,000-step BH simulation of the experimental structure with charged termini, using CS and DC constraints. The results include a bend in the helix, highlighting the necessity to cap the terminal groups, which were introduced using LEaP^61^ for AMBER.

**Figure 2:**
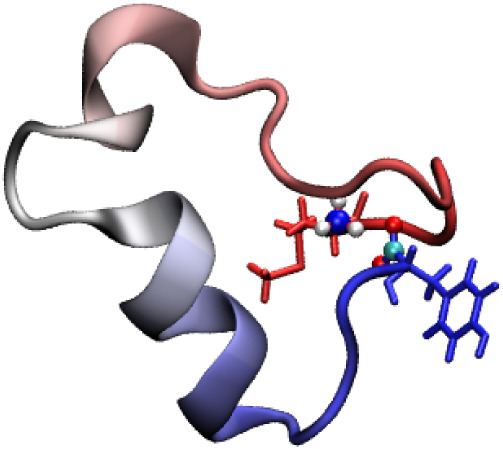
SLN BH results with charged termini optimized using a hybrid potential (AMBER ff19SB force field coupled with DC and CS constraints). Red and blue colors denote N- and C-terminal regions, respectively. The first (methionine) and last (tyrosine) residues are shown explicitly with the charged amide and carboxyl ends, respectively.

#### 3.1.1 Structure Refinement

The starting point for our studies is the experimentally determined NMR structure of SLN (PDB ID: 1JDM),^62^ capped at both ends, as described above. The principal axis of the SLN helix is aligned perpendicular to the membrane bilayer normal vector, parallel to the external magnetic field. Table 1 shows the lowest BH minimum results for the CS and DC constrained potentials interfaced with AMBER ff19SB and CHARMM36m force fields (termed AMBER and CHARMM for simplicity).

**Table 1:** Energy Breakdown and Order Parameters for the Lowest Energy Structures Obtained from BH Runs Performed with Structurally and Orientationally Constrained Potentials, i.e., CS and DC Constraints Coupled with Force Field Potentials

BH results	AMBER	CHARMM
*V*_Total_ (kcal mol^–1^)	–1197.7225	–520.3033
*V*_CS_ (kcal mol^–1^)	3.6215	20.1205
*V*_DC_ (kcal mol^–1^)	2.0013	6.3225
Tilt Angle (°)	32.4	32.0
*N*_b_	16/16	14/16

Our results suggest that AMBER is a better choice for studying SLN in the current setup. AMBER was observed to support more native-like structures with higher helical propensities under similar NapShift contributions. Figure 3 shows the Ramachandran plots for the CS and DC constrained structures obtained using (a) AMBER and (b) CHARMM. AMBER supports more residues with α-helical structure, corresponding to the number of residues in the lower left blue region. We therefore used the AMBER force field for further simulations on SLN. Our structure refinement studies demonstrate the effectiveness of using NMR constraints within the hybrid potential setup to describe the membrane environment.

**Figure 3:**
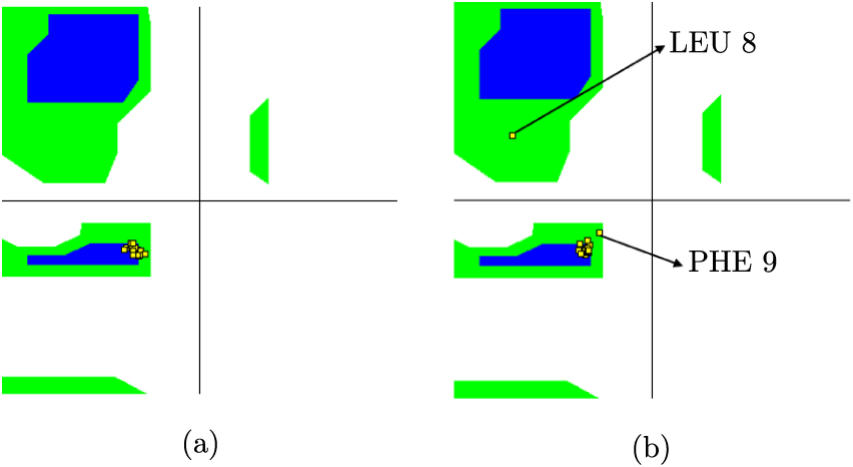
Ramachandran plots for optimized SLN structures obtained using constrained (a) AMBER and (b) CHARMM. Only the helical portion has been shown here. LEU8 and PHE9 do not fall in the α-helical region with CHARMM.

#### 3.1.2 Structure Prediction

Having determined that the NMR hybrid potential provides a useful description of the membrane environment for SLN, the next step is to study the feasibility of these hybrid potentials in reaching the native state of SLN starting from a fully extended chain of amino acids, i.e., beginning with no secondary structural element. The starting configuration was further oriented to a fixed angle of 90° with respect to the *z*-axis, which is the direction of the membrane bilayer normal. For this purpose, all studies were carried out without any additional implicit or explicit solvent and membrane, as for the structure refinement procedure. A fully extended structure, shown in Figure 4a, was used as the starting configuration. AMBER was considered as the baseline force field, as explained in the previous section.

**Figure 4:**
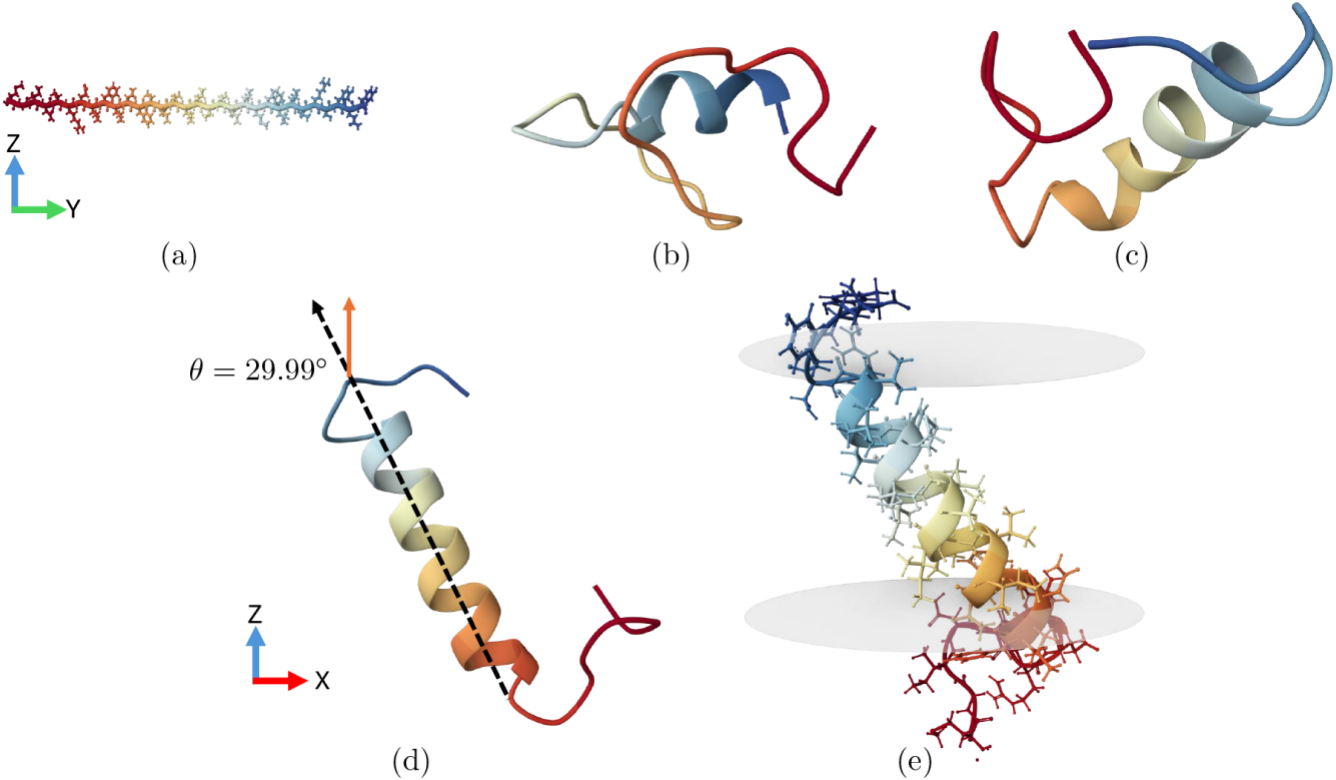
Structure prediction results for SLN. (a) Fully extended chain of amino acids, used as the starting structure. (b) Lowest energy minimum obtained with unconstrained AMBER in 100,000 BH steps. (c) Lowest energy minimum obtained with restraints derived from synthetic CS in 50,000 BH steps. This potential represents an intermediate step in our hierarchical optimization procedure. (d) Global minimum obtained with restraints derived from synthetic CS and experimental DC data. (e) Experimental structure for reference, where leaflets represent lipid membranes.

To begin with, AMBER was employed on its own for global optimization. The lowest-energy minimum obtained using this standalone force field within 100,000 BH steps is presented in Figure 4b. In the absence of any external stabilizing environment, the standard BH run does not escape from this low energy funnel where the structure deviates from the native protein conformation, as indicated by the nonhelical TM region. This observation suggests a frustrated energy landscape with competing funnels and highlights the difficulty in reaching the native state using the standalone AMBER force field in the absence of a solvent and membrane representation. We seek a remedy for this problem with the hybrid potential.

Starting from the fully extended structure, CS restraints were then introduced in the hybrid potential using NapShift. Owing to the paucity of experimental data for several atoms in the helical region, we utilized the predicted chemical shifts from NapShift (referred to as synthetic CS), corresponding to the NMR structure of SLN. The synthetic CS are listed in the Supporting Information. Thus, beginning from the extended structure, a new BH search was executed for 50,000 steps, with error tolerance (ϵ) set to 0.25. The contribution from the CS penalty energy was set to approximately 10% of the total energy, and was gradually decreased as the structure began to align with the experimental geometry. The lowest energy structure obtained is shown in Figure 4c, and it largely resembles the experimentally obtained native structure, shown in Figure 4e. Furthermore, because this structure is intermediary in our hierarchical approach, the global minimum conformation was not required. Additionally, since CS values do not have any orientational significance associated with them, the angle that the current structure makes with the *z*-axis is arbitrary. Here, the only steps responsible for varying the orientation are the randomly chosen dihedral moves. This setup highlighted the need to incorporate DC based orientational restraints in our study. The CS restrained hybrid potential induces large penalty energies for the experimentally incompatible conformations and lowers the barrier heights involved in forming the correct structure. This transformation, in turn, results in efficient and easier identification of the native-like conformations. The results illustrate the importance of a rich set of NMR data as a prerequisite for using hybrid potentials.

Next, the configuration in Figure 4c was used as the starting structure for the structurally and orientationally constrained potential (AMBER + CS + DC). The DC data employed in this work are summarized in the Supporting Information. Fully constrained BH simulations were run for a total of 50,000 steps at temperatures ranging from 1.0 to 2.0 kcal mol^–1^ in energy units. The contribution of DC to the total energy was adjusted using a weight parameter (α_DC_), which was varied between 5 and 10, and the error tolerance was set to 0.1 kHz. The CS contribution was maintained to provide the necessary structural information.

The global minimum obtained for the fully constrained case is shown in Figure 4d, with a tilt angle of 29.9°. Tilt angles were monitored at regular intervals for two cases: standalone AMBER and the AMBER + CS + DC hybrid potential. Figure 5 presents the progression of tilt angles across the two BH simulations. The orientational constraint (DC) was introduced in a hierarchical manner after 50,000 BH steps within the hybrid potential. Convergence to the correct tilt angle was achieved on inclusion of the DC constraint. Table 2 highlights the lowest energy structures’ RMSD values and energy breakdown for different potentials studied here.

**Figure 5:**
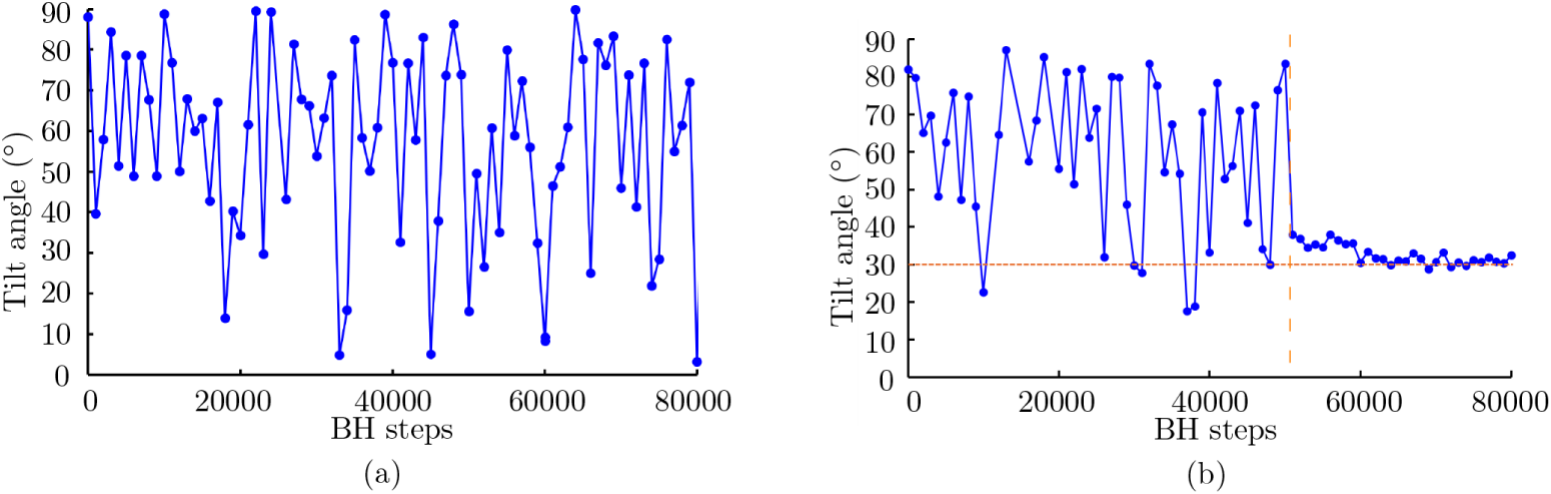
Progression of the TM tilt angle of the (a) unconstrained and (b) constrained basin-hopping runs for SLN. The dashed horizontal orange line in (b) indicates the best fitted tilt angle for the DC constrained steps. In (b), DC was applied after 50,000 BH steps, indicated by the dashed vertical orange line, following the formation of the correct helix.

**Table 2:** Analysis of Lowest Energy Structures from BH Runs Performed on SLN with Hybrid Restraint Potentials, Starting with the Fully Extended Structure^a^

Potential	*V*_Total_ (kcal mol^–1^)	*V*_CS_ (kcal mol^–1^)	*V*_DC_ (kcal mol^–1^)	RMSD (Å)
**AMBER**	–1209.8402	N/A	N/A	8.7566
**AMBER + CS**	–1182.8630	11.0751	N/A	3.1567
**AMBER + CS + DC**	–1195.7137	2.7924	3.6948	1.1222

a RMSD values correspond to the TM region (residues 7–16) with respect to the refined experimental structure.

The DC-constrained results indicate an average tilt angle of around 30°, which is in close agreement with values reported in the literature. Thus, these results from studies on SLN illustrate the effectiveness of using NMR observables in structure prediction and refinement for membrane proteins, where they define an effective environment.

#### 3.1.3 Folding Pathways and Energy Landscapes

In this section, we investigate the potential energy landscapes associated with the SLN folding pathway, i.e., the transition from the fully extended structure to the fully formed MP helical structure. We compare the pathway obtained with the standalone AMBER force field against that produced by our hybrid potential setup, and further analyze the influence of varying the weight of the CS constraint. No orientational constraint is applied in this analysis. The resulting pathways are visualized using disconnectivity graphs, as shown in Figure 6.

**Figure 6:**
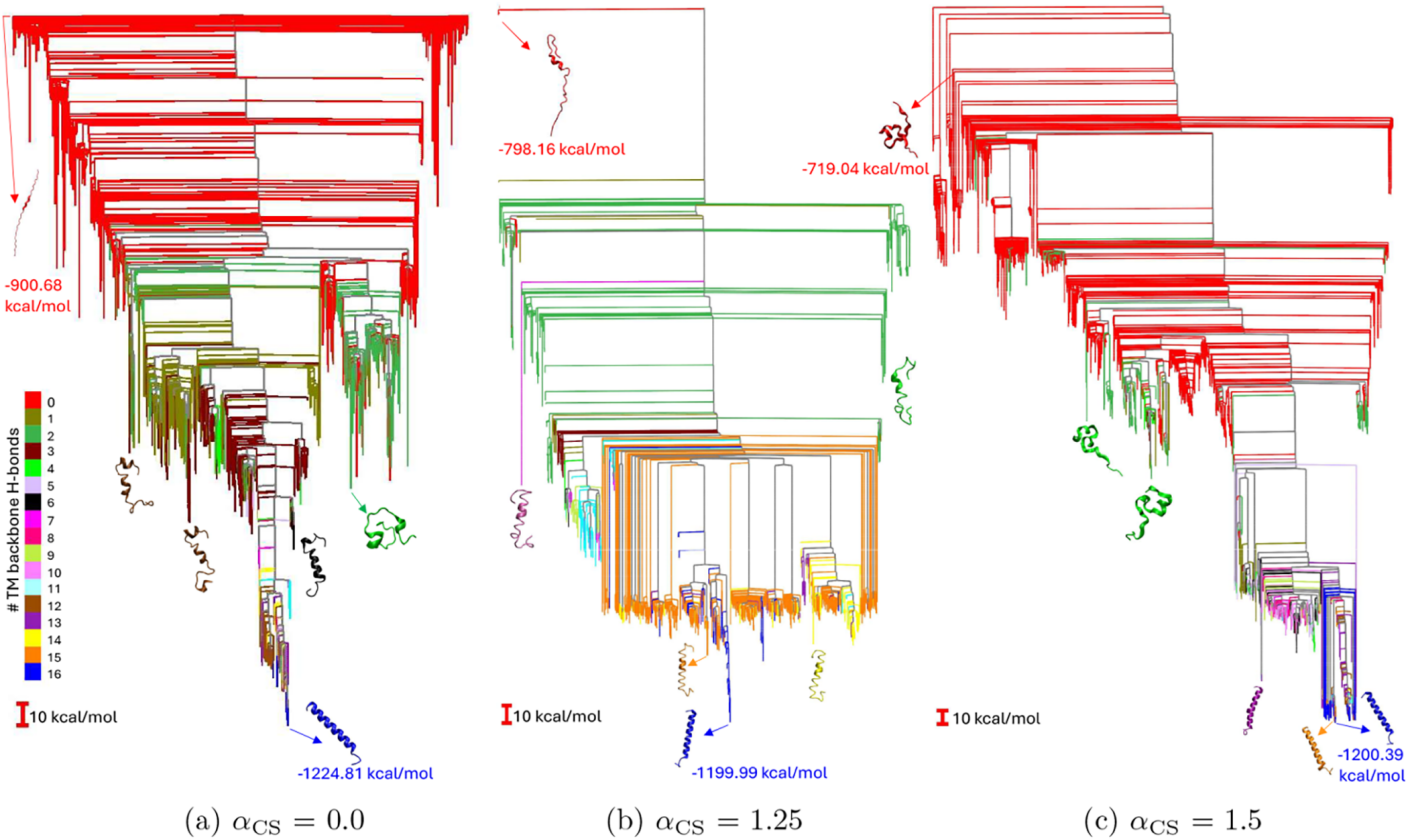
Disconnectivity graphs based on the folding pathways for SLN with hybrid potential mixing parameter values of (a) α_CS_ = 0.0, (b) α_CS_ = 1.25, and (c) α_CS_ = 1.5. The color scheme is based on the number of native hydrogen bonds in the transmembrane region and is given by the scale on the left. The structures shown for each disconnectivity graph correspond to minima from the corresponding energy landscape.

The α_CS_ = 0.0 result represents the unconstrained AMBER potential, where the lowest energy structure was identified during refinement starting from the experimental structure. The introduction of CS constraints raises the energy of the unfolded conformations because they are very different from the NMR structure, and therefore incur high positive CS penalty energies. The penalty energy progressively increases as we increase the CS constraint, tuned with the mixing parameter α_CS_, and further penalizes the experimentally incompatible structures. Disconnectivity graphs illustrating these trends are colored according to the order parameter *N*_b_. The unconstrained case consists of multiple badly formed helices in the lowest energy funnel. The α_CS_ = 1.25 result produces the ideal case where the native structure is stabilized the most and the experimentally incompatible structures are raised high in energy. The lowest energy funnel consists of many more near-native structures, compared to the unconstrained case. However, addition of constraints needs to be in a certain range. A higher value, α_CS_ = 1.5, produces overfitting to the NMR data, where the low lying funnel supports multiple competing structures. We also observe some unphysical structures, shown in orange, which are well fitted to the NMR data but have higher AMBER potential energy. Thus, α_CS_ = 1.25 represents the ideal value to study the folding mechanism of SLN using this hybrid potential setup.

While exploring the CS restrained potential energy landscapes, we encountered a challenge arising from the fact that NapShift is not invariant to permutational symmetry. As a result, the database consisting of minima and transition states included structures that are equivalent under permutation within experimental time scales. This issue was particularly evident in leucine and valine residues where methyl group rotations led to unphysical sp^3^ inversion pathways, making it difficult to find a connected folding pathway for SLN. To remedy this problem, we checked the database and manually swapped the coordinates of the symmetric groups in the affected residues, as required, to ensure consistency. This correction was followed by LBFGS relaxation, and additional OPTIM and PATHSAMPLE jobs were run to regenerate connections and subsequently merge the databases. Such artifacts would not arise if NapShift were permutationally symmetric; work to implement this symmetry is currently in progress.

### 3.2 Transmembrane Protein System 2: Phospholamban

To further evaluate the effectiveness of the hybrid potential, we next examine a larger transmembrane protein, phospholamban (PLN). PLN is a 53-residue mammalian protein, with sequence AMEKVQYLTRSAIRRASTIEMPQQARQNLQNLFINFALILIFLLLIAIIVMLL. It is located in the sarcoplasmic reticulum of cardiac and smooth muscles,^63^ and regulates cardiac contractility by forming pentamers that dissociate into active monomers, which then inhibit the Ca^2+^ pump through electrostatic interactions.^63,64^ The monomer consists of four subdomains: Ia (residues 1–16), loop (17–22), Ib (23–30), and II (31–52). It exists in equilibrium between a dynamically disordered R-state and a more restricted T-state, the latter representing the resting form and accounting for 84% of the population.^65^ PLN therefore represents a more complex case, characterized by an underlying multifunneled landscape. In the following section, we employ optimized hybrid restraint potentials to study the conformational dynamics of monomeric PLN, again in the absence of a conventional explicit or implicit representation for the membrane environment.

#### 3.2.1 R- to T-State Conformational Analysis

Starting from the capped NMR structure of the R-state (PDB ID 2LPF),^66^ global optimization was carried out to bias the conformational search toward the native T-state, as depicted in Figure 7. For this purpose, experimental NMR data corresponding to the T-state were employed. CS constraints (derived from BMRB entry 50718), were introduced to encourage formation and stability of alpha helical conformations and to represent the lipid and water molecules. CSA and DC data (listed in the SI) were used as orientational constraints to support native-like angular separations corresponding to the T-state. ssNMR experiments have shown that regions Ib and II together form a transmembrane helix with a tilt angle of around 24° to the positive *z*-axis, and an angle of 107° between the two helices.^59^ We also made use of NOE distances in the hybrid potential to discourage artificial helical–helical interactions, which are mostly force field induced. Our preliminary studies suggest that the CHARMM force field is suitable for preventing overfitting to the experimental constraints in the current hybrid potential setup for PLN. The relative contributions from these constraints were varied hierarchically, making sure that orientational biasing succeeded secondary structural biasing in order. RMS convergence criteria were kept unchanged as above. Table 3 includes BH results for the hybrid potential global minimum. This approach allowed us to reach the near-native structure and orientation within 80,000 BH steps. These results indicate that combining BH global optimization with a hybrid potential that incorporates multiple NMR constraints to represent the environment can successfully identify alternative physically relevant pathways for conformational changes.

**Figure 7:**
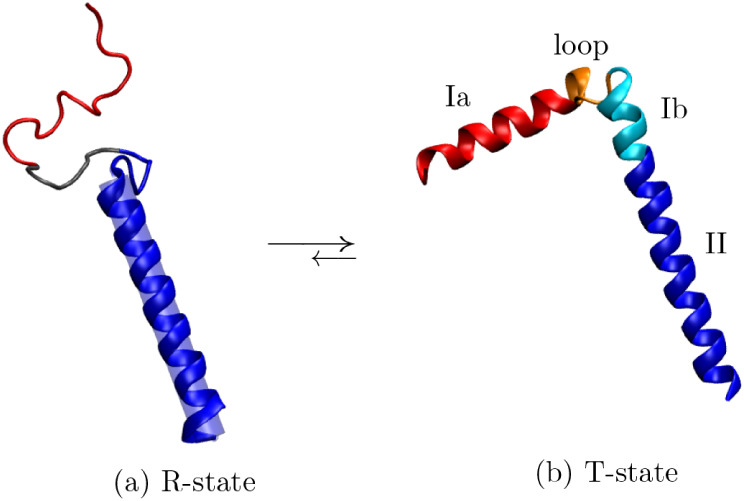
(a) Starting (R-state) and (b) target (T-state) structures for the conformational change. The longer forward arrow indicates that the forward transition is faster.

**Table 3:** BH Results for the PLN Conformational Transition from the R-State to the T-State, Including the Energy Decomposition (in kcal mol^–1^) and the Backbone RMSD Relative to the Reference T-State

*V*_Total_	*V*_CHARMM_	*V*_CS_	*V*_CSA_	*V*_DC_	*V*_DIST_	RMSD (Å)	Tilt Angle (°)	Interhelix Angle (°)
–1267.57	–1297.87	11.03	9.56	7.78	1.93	0.592	24.7	106.1

Further analysis was carried out on the conformational change between the R- and T-states using discrete path sampling. Figure 8 shows the pathway contributing most significantly to the steady-state rate at 300 K. Intervening minima shed light on the structural and orientational changes that occur in the process. The second helix formed first under the influence of CS restraints, after which orientational restraints induced gradual adjustments and slight distortions in the TM helix to achieve the correct orientation.

**Figure 8:**
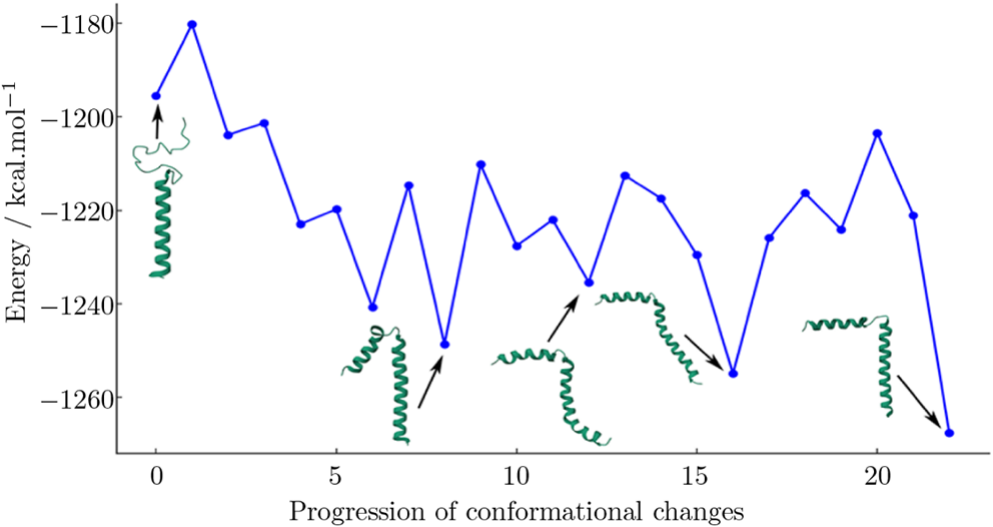
Energy profile of the conformational transition from the R-state to the T-state of PLN, shown in terms of selected intervening minima. The vertical axis shows the hybrid energy, while the horizontal axis indicates the progression through these stationary points. Selected intermediate minima are labeled in the graph.

## 4 Conclusions

In this study, we explored the utility of NMR data to study membrane proteins by developing hybrid restraint potentials. Different NMR observables, namely, chemical shift, dipolar coupling, chemical shift anisotropy, and NOE distances were employed to constrain basin-hopping global optimization and discrete path sampling for two transmembrane protein systems, namely, sarcolipin and phospholamban. Our results illustrate the capabilities of the hybrid potential setup, in supporting near-native structural and orientational conformations for membrane proteins with no other explicit/implicit environments.

Chemical shift constraints were observed to improve the secondary structure formation for sarcolipin and phospholamban, and stabilize the TM structure in the absence of explicit/implicit lipid and water molecules. Dipolar coupling and chemical shift anisotropy constraints were found to be sufficient to produce an experimentally compatible orientation. Starting from a fully extended structure of sarcolipin, chemical shift and dipolar coupling constraints were found to bring the structure and orientation into agreement with experimental NMR data. The impact of the mixing parameters on the underlying potential energy landscape for SLN folding pathway was also explored. We find that a balance between dipolar coupling, chemical shift anisotropy, and NOE distance restraints in the hybrid potential setup is required to guide the PLN orientation toward experimentally compatible structures, and produce well-funneled landscapes, where the native state is thermodynamically stable and kinetically accessible. PLN conformational change was also analyzed. Moving forward, we aim to extend this approach to study the R-state and pentameric state of PLN, for which NMR data have become available.

Overall, constrained cases were associated with greater experimental compatibility compared to the unconstrained force field case, and allowed us to reach near-native states in fewer optimization steps. Although this is the expected result, we find that optimal weights for different constraints are system and case specific; thus, preliminary surveys are required to obtain a good hybrid potential setup for any new system. These weights are bounded between zero, corresponding to an unconstrained force-field potential, and large values at which overfitting to the NMR data occurs. Based on our experience across a range of systems, a practical starting point is a regime in which the NMR restraints contribute approximately 10–20% of the total potential energy. This initial choice is then refined by testing nearby values. The optimal value of weights is assessed by examining the resulting energy landscapes. The aim is to obtain a well-funneled and minimally frustrated landscape consistent with the NMR data.

Having validated our approach for transmembrane α-helices, extending this framework to more complex β-sheet systems is a future research goal. Based on our previous studies, we expect the methodology to be useful for β-barrel membrane proteins and systems with more complex topologies, where experimental challenges often arise from the lack of simple axial symmetry and high chemical shift degeneracy. In addition, application of the NMR hybrid restraint potential to larger viral membrane proteins with available experimental NMR data is currently underway and will be discussed in a future report.

## Supplementary Material



## Notes

NMR data for the membrane proteins discussed in this paper; the GMIN,^26^ OPTIM,^27^ and PATHSAMPLE^51^ (PDF)
