# Supplementary material for: Energy Landscape Analysis of Membrane Proteins Using NMR-Based Hybrid Restraint Potentials
Source: J Chem Theory Comput. 2026 Mar 12;22(11):5827–37. doi: 10.1021/acs.jctc.5c02070 (PMC13255242; doi:10.1021/acs.jctc.5c02070)
Supplement: Supplementary file 1 [file ct5c02070_si_001.pdf]

Supporting Information

Energy Landscape Analysis of Membrane Proteins Using NMR-Based Hybrid Restraint Potentials

Diksha Dewan,[1] Yifei Wang,[1] Alfonso De Simone,[2] and David J. Wales[1,*]

[1] *Yusuf Hamied Department of Chemistry, University of Cambridge, Lensfield Road, Cambridge, CB2 1EW, U.K.*

[2] *Department of Pharmacy, University of Naples Federico II, Naples, Italy*

I. Chemical Shift Data for Sarcolipin

Table S1: Secondary CS data, in ppm, corresponding to the primary experimental CS data derived from Ref [1].

RES ID	Residue	$^1H_\alpha$	$^{13}C_\alpha$	^1H	^{15}N	^{13}C	$^{13}C_\beta$
1	ACE	0.0000	0.0000	0.0000	0.0000	0.0000	0.0000
2	MET	0.0000	0.0000	0.0000	0.0000	0.0000	0.0000
3	GLY	-0.1180	0.0000	0.2244	0.0000	0.0000	0.0000
4	ILE	0.0947	0.0000	-0.5380	0.0000	0.0000	0.0000
5	ASN	0.1170	0.0000	-0.3023	0.0000	0.0000	0.0000
6	THR	-0.1334	0.0000	-0.0698	0.0000	0.0000	0.0000
7	ARG	-0.1622	0.0000	-0.1878	0.0000	0.0000	0.0000
8	GLU	-0.1641	0.0000	-0.5701	0.0000	0.0000	0.0000
9	LEU	-0.1898	0.0000	-0.0776	0.0000	0.0000	0.0000
10	PHE	-0.2306	0.0000	0.0129	0.0000	0.0000	0.0000
11	LEU	-0.2425	0.0000	0.1977	0.0000	0.0000	0.0000
12	ASN	0.1973	0.0000	-0.1788	0.0000	0.0000	0.0000
13	PHE	-0.5076	0.0000	0.3130	0.0000	0.0000	0.0000
14	THR	-0.8451	0.0000	-0.2272	0.0000	0.0000	0.0000
15	ILE	-0.5783	0.0000	-0.4220	0.0000	0.0000	0.0000
16	VAL	-0.7025	0.0000	-0.3002	0.0000	0.0000	0.0000
17	LEU	-0.5562	0.0000	0.0278	0.0000	0.0000	0.0000
18	ILE	-0.6380	0.0000	0.0570	0.0000	0.0000	0.0000

* Corresponding author: dw34@cam.ac.uk

19	THR	-0.7410	0.0000	-0.2004	0.0000	0.0000	0.0000
20	VAL	-0.3512	0.0000	0.1036	0.0000	0.0000	0.0000
21	ILE	-0.5838	0.0000	-0.0078	0.0000	0.0000	0.0000
22	LEU	-0.2723	0.0000	0.4294	0.0000	0.0000	0.0000
23	MET	-0.3179	0.0000	0.3898	0.0000	0.0000	0.0000
24	TRP	-0.4281	0.0000	0.7229	0.0000	0.0000	0.0000
25	LEU	-0.6592	0.0000	0.5900	0.0000	0.0000	0.0000
26	LEU	-0.2419	0.0000	0.5657	0.0000	0.0000	0.0000
27	VAL	-0.4390	0.0000	0.5389	0.0000	0.0000	0.0000
28	ARG	-0.3376	0.0000	0.0715	0.0000	0.0000	0.0000
29	SER	-0.2127	0.0000	-0.4175	0.0000	0.0000	0.0000
30	TYR	-0.1389	0.0000	-0.2544	0.0000	0.0000	0.0000
31	GLN	-0.0621	0.0000	-0.4519	0.0000	0.0000	0.0000
32	TYR	0.0000	0.0000	0.0000	0.0000	0.0000	0.0000
33	NME	0.0000	0.0000	0.0000	0.0000	0.0000	0.0000

Table S2: Synthetic CS data, in ppm, for SLN.

RES ID	Residue	$^{1}H_{\alpha}$	$^{13}C_{\alpha}$	^{1}H	^{15}N	^{13}C	$^{13}C_{\beta}$
1	ACE	0.0000	0.0000	0.0000	0.0000	0.0000	0.0000
2	MET	0.0000	0.0000	0.0000	0.0000	0.0000	0.0000
3	GLY	0.0000	0.0000	0.0000	0.0000	0.0000	0.0000
4	ILE	0.0000	0.0000	0.0000	0.0000	0.0000	0.0000
5	ASN	0.0000	0.0000	0.0000	0.0000	0.0000	0.0000
6	THR	0.0000	0.0000	0.0000	0.0000	0.0000	0.0000
7	ARG	0.0000	0.0000	0.0000	0.0000	0.0000	0.0000
8	GLU	-0.1567	0.5252	0.1029	-2.1259	0.2902	-0.2422
9	LEU	-0.0232	-0.0579	-0.1303	-4.1006	-0.4182	-0.8536
10	PHE	-0.5022	2.5784	-0.3892	-0.9368	0.8765	-1.1101
11	LEU	-0.5852	2.6181	0.0524	-3.3688	1.4050	-1.4832
12	ASN	-0.3359	2.3891	-0.2851	-3.3425	2.0635	-0.8699
13	PHE	-0.5039	3.2014	0.1831	-1.0291	0.5943	-0.9974
14	THR	-0.6354	4.7200	-0.0708	-0.0675	1.5552	-1.6849
15	ILE	-0.5303	3.6954	-0.5679	-1.9453	1.2763	-0.8967
16	VAL	-0.5905	3.7214	-0.1623	-5.0021	1.7470	-1.5043
17	LEU	-0.4205	2.1021	-0.3368	-6.3709	1.0408	-0.6420

18	ILE	-0.4484	3.0778	-0.2408	-5.3159	0.7242	-1.5267
19	THR	-0.5094	4.6598	-0.2845	-2.2004	1.1495	-1.5321
20	VAL	-0.6809	4.3272	-0.1536	-1.0785	0.9247	-1.5122
21	ILE	-0.6111	3.7430	-0.4233	-5.5204	1.2055	-1.4307
22	LEU	-0.4962	1.7243	-0.1372	-6.0053	0.7522	-1.1194
23	MET	-0.5220	2.5578	-0.2143	-3.8157	1.5820	-0.1886
24	TRP	-0.4876	2.2330	0.0694	-1.9545	0.4260	-1.0879
25	LEU	-0.6308	1.1822	-0.3828	-5.5408	0.1744	-0.3491
26	LEU	-0.2832	1.5331	-0.2306	-4.1152	0.7846	-0.5651
27	VAL	-0.5557	3.0791	-0.1925	-3.0812	0.7053	-1.0953
28	ARG	0.0000	0.0000	0.0000	0.0000	0.0000	0.0000
29	SER	0.0000	0.0000	0.0000	0.0000	0.0000	0.0000
30	TYR	0.0000	0.0000	0.0000	0.0000	0.0000	0.0000
31	GLN	0.0000	0.0000	0.0000	0.0000	0.0000	0.0000
32	TYR	0.0000	0.0000	0.0000	0.0000	0.0000	0.0000
33	NME	0.0000	0.0000	0.0000	0.0000	0.0000	0.0000

II. Dipolar Coupling Data for Sarcolipin

The GMIN and OPTIM implementation of DC restraint requires an input file, called dipolarcoupling, which contains DC values in kHz. Below we show the format of the input file. ID is 1 for N-H atom pair and 3 for C_α-H_α atom pair.

Table S3: Format for *dipolarcoupling*, a file required for GMIN and OPTIM implementation of DC restraint.

ID	Atom Number	DC_{NH}/kHz	ϵ_{DC}/kHz	ξ_{DC}/kHz
1	78	6.90	0.1	10.52
1	102	5.80	0.1	10.52
1	117	3.20	0.1	10.52
1	136	4.80	0.1	10.52
1	156	8.00	0.1	10.52
1	175	6.10	0.1	10.52
1	189	3.00	0.1	10.52
1	209	6.90	0.1	10.52
1	223	6.40	0.1	10.52
1	242	3.10	0.1	10.52
1	258	4.70	0.1	10.52

1	277	7.80	0.1	10.52
1	296	3.90	0.1	10.52
1	310	2.90	0.1	10.52
1	326	6.10	0.1	10.52
1	345	7.50	0.1	10.52
1	364	3.00	0.1	10.52
1	381	4.50	0.1	10.52
1	405	6.90	0.1	10.52
1	424	5.30	0.1	10.52
1	443	3.60	0.1	10.52
1	459	7.90	0.1	10.52
1	483	5.00	0.1	10.52
1	494	2.60	0.1	10.52
1	515	2.60	0.1	10.52
1	532	2.00	0.1	10.52

III. Dipolar Coupling Data for Phospholamban

Table S4: Dipolar coupling data for Phospholamban.

ID	Atom Number	DC_{NH}/kHz	ϵ_{DC}/kHz	ξ_{DC}/kHz
1	45	4.900	0.5	10.52
1	83	0.000	0.5	10.52
1	100	4.600	0.5	10.52
1	121	5.100	0.5	10.52
1	140	3.500	0.5	10.52
1	154	3.500	0.5	10.52
1	178	5.100	0.5	10.52
1	189	0.000	0.5	10.52
1	199	3.500	0.5	10.52
1	219	3.700	0.5	10.52
1	242	5.200	0.5	10.52
1	266	3.000	0.5	10.52
1	276	5.100	0.5	10.52
1	287	0.000	0.5	10.52
1	383	6.200	0.5	10.52
1	410	8.500	0.5	10.52

1	434	5.700	0.5	10.52
1	451	4.800	0.5	10.52
1	465	8.200	0.5	10.52
1	484	6.500	0.5	10.52
1	501	4.000	0.5	10.52
1	515	7.100	0.5	10.52
1	534	8.000	0.5	10.52
1	554	6.100	0.5	10.52
1	573	4.100	0.5	10.52
1	587	7.200	0.5	10.52
1	607	8.200	0.5	10.52
1	617	4.100	0.5	10.52
1	636	6.600	0.5	10.52
1	655	8.300	0.5	10.52
1	674	7.600	0.5	10.52
1	693	4.300	0.5	10.52
1	713	7.300	0.5	10.52
1	732	8.200	0.5	10.52
1	751	4.100	0.5	10.52
1	770	6.200	0.5	10.52
1	789	8.300	0.5	10.52
1	799	7.400	0.5	10.52
1	818	4.600	0.5	10.52
1	837	6.100	0.5	10.52
1	853	8.300	0.5	10.52
1	870	4.000	0.1	10.52

IV. Chemical Shift Anisotropy Data for Phospholamban

The GMIN and OPTIM implementation of CSA restraint requires an input file, called ssnmr.dat, which contains CSA values in ppm. Below we show the format of the input file. ID is set to 0 for CSA to differentiate it from other restraints.

Table S5: Chemical shift anisotropy data for phospholamban in the format required by the GMIN and OPTIM implementation of CSA restraint.

ID	First Atom	Second Atom	Third Atom	CSA/ppm	Error tolerance/ppm	Tensor magnitude_11/ppm	Tensor magnitude_22/ppm	Tensor magnitude_33/ppm

0	45	46	44	71.000	5.0	64.0	76.0	216.9
0	83	84	82	79.000	5.0	64.0	76.0	216.9
0	100	101	99	78.000	5.0	64.0	76.0	216.9
0	121	122	120	79.000	5.0	64.0	76.0	216.9
0	140	141	139	77.000	5.0	64.0	76.0	216.9
0	154	155	153	84.000	5.0	64.0	76.0	216.9
0	178	179	177	63.000	5.0	64.0	76.0	216.9
0	189	190	188	77.000	5.0	64.0	76.0	216.9
0	199	200	198	85.000	5.0	64.0	76.0	216.9
0	219	218	217	79.000	5.0	64.0	76.0	216.9
0	242	243	241	69.000	5.0	64.0	76.0	216.9
0	266	267	265	81.000	5.0	64.0	76.0	216.9
0	276	277	275	77.000	5.0	64.0	76.0	216.9
0	287	288	286	65.000	5.0	64.0	76.0	216.9
0	383	384	382	214.000	5.0	64.0	76.0	216.9
0	410	411	409	209.000	5.0	64.0	76.0	216.9
0	434	435	433	164.000	5.0	64.0	76.0	216.9
0	451	452	450	199.000	5.0	64.0	76.0	216.9
0	465	466	464	218.000	5.0	64.0	76.0	216.9
0	484	485	483	168.000	5.0	64.0	76.0	216.9
0	501	502	500	187.000	5.0	64.0	76.0	216.9
0	515	516	514	208.000	5.0	64.0	76.0	216.9
0	534	535	533	167.000	5.0	64.0	76.0	216.9
0	554	555	553	161.000	5.0	64.0	76.0	216.9
0	573	574	572	198.000	5.0	64.0	76.0	216.9
0	587	588	586	223.000	5.0	64.0	76.0	216.9
0	607	608	606	177.000	5.0	64.0	76.0	216.9
0	617	618	616	167.000	5.0	64.0	76.0	216.9
0	636	637	635	217.000	5.0	64.0	76.0	216.9
0	655	656	654	180.000	5.0	64.0	76.0	216.9
0	674	675	673	163.000	5.0	64.0	76.0	216.9
0	693	694	692	194.000	5.0	64.0	76.0	216.9
0	713	714	712	217.000	5.0	64.0	76.0	216.9
0	732	733	731	181.000	5.0	64.0	76.0	216.9
0	751	752	750	167.000	5.0	64.0	76.0	216.9
0	770	771	769	215.000	5.0	64.0	76.0	216.9

0	789	790	788	191.000	5.0	64.0	76.0	216.9
0	799	800	798	158.000	5.0	64.0	76.0	216.9
0	818	819	817	180.000	5.0	64.0	76.0	216.9
0	837	838	836	214.000	5.0	64.0	76.0	216.9
0	853	854	852	185.000	5.0	64.0	76.0	216.9
0	870	871	869	184.000	5.0	64.0	76.0	216.9

V. NOE Distance Data for Phospholamban

All distances are in Angstrom. Error tolerance and harmonic constant are set to 1.0 Å and 1.0 kcal/mol, respectively. The lines below define the atom pairs and provide three numbers that indicate the target inter-atomic distance, as well as the corresponding lower and upper bounds.

assign (resid 4 and name O) (resid 8 and name HN) 1.90 0.40 0.40
assign (resid 8 and name N) (resid 4 and name O) 2.85 0.55 0.55
!
assign (resid 5 and name O) (resid 9 and name HN) 1.90 0.40 0.40
assign (resid 9 and name N) (resid 5 and name O) 2.85 0.55 0.55
!
assign (resid 6 and name O) (resid 10 and name HN) 1.90 0.40 0.40
assign (resid 10 and name N) (resid 6 and name O) 2.85 0.55 0.55
!
assign (resid 7 and name O) (resid 11 and name HN) 1.90 0.40 0.40
assign (resid 11 and name N) (resid 7 and name O) 2.85 0.55 0.55
!
assign (resid 8 and name O) (resid 12 and name HN) 1.90 0.40 0.40
assign (resid 12 and name N) (resid 8 and name O) 2.85 0.55 0.55
!
assign (resid 9 and name O) (resid 13 and name HN) 1.90 0.40 0.40
assign (resid 13 and name N) (resid 9 and name O) 2.85 0.55 0.55
!
assign (resid 10 and name O) (resid 14 and name HN) 1.90 0.40 0.40
assign (resid 14 and name N) (resid 10 and name O) 2.85 0.55 0.55
!
assign (resid 24 and name O) (resid 28 and name HN) 1.90 0.40 0.40
assign (resid 28 and name N) (resid 24 and name O) 2.85 0.55 0.55

```
!
assign (resid 25 and name O ) (resid 29 and name HN ) 1.90 0.40 0.40
assign (resid 29 and name N ) (resid 25 and name O ) 2.85 0.55 0.55
!
assign (resid 26 and name O ) (resid 30 and name HN ) 1.90 0.40 0.40
assign (resid 30 and name N ) (resid 26 and name O ) 2.85 0.55 0.55
!
assign (resid 27 and name O ) (resid 31 and name HN ) 1.90 0.40 0.40
assign (resid 31 and name N ) (resid 27 and name O ) 2.85 0.55 0.55
!
assign (resid 28 and name O ) (resid 32 and name HN ) 1.90 0.40 0.40
assign (resid 32 and name N ) (resid 28 and name O ) 2.85 0.55 0.55
!
assign (resid 29 and name O ) (resid 33 and name HN ) 1.90 0.40 0.40
assign (resid 33 and name N ) (resid 29 and name O ) 2.85 0.55 0.55
!
assign (resid 30 and name O ) (resid 34 and name HN ) 1.90 0.40 0.40
assign (resid 34 and name N ) (resid 30 and name O ) 2.85 0.55 0.55
!
assign (resid 31 and name O ) (resid 35 and name HN ) 1.90 0.40 0.40
assign (resid 35 and name N ) (resid 31 and name O ) 2.85 0.55 0.55
!
assign (resid 32 and name O ) (resid 36 and name HN ) 1.90 0.40 0.40
assign (resid 36 and name N ) (resid 32 and name O ) 2.85 0.55 0.55
!
assign (resid 33 and name O ) (resid 37 and name HN ) 1.90 0.40 0.40
assign (resid 37 and name N ) (resid 33 and name O ) 2.85 0.55 0.55
!
assign (resid 34 and name O ) (resid 38 and name HN ) 1.90 0.40 0.40
assign (resid 38 and name N ) (resid 34 and name O ) 2.85 0.55 0.55
!
assign (resid 35 and name O ) (resid 39 and name HN ) 1.90 0.40 0.40
assign (resid 39 and name N ) (resid 35 and name O ) 2.85 0.55 0.55
```

!

assign (resid 36 and name O) (resid 40 and name HN) 1.90 0.40 0.40

assign (resid 40 and name N) (resid 36 and name O) 2.85 0.55 0.55

!

assign (resid 37 and name O) (resid 41 and name HN) 1.90 0.40 0.40

assign (resid 41 and name N) (resid 37 and name O) 2.85 0.55 0.55

!

assign (resid 38 and name O) (resid 42 and name HN) 1.90 0.40 0.40

assign (resid 42 and name N) (resid 38 and name O) 2.85 0.55 0.55

!

assign (resid 39 and name O) (resid 43 and name HN) 1.90 0.40 0.40

assign (resid 43 and name N) (resid 39 and name O) 2.85 0.55 0.55

!

assign (resid 40 and name O) (resid 44 and name HN) 1.90 0.40 0.40

assign (resid 44 and name N) (resid 40 and name O) 2.85 0.55 0.55

!

assign (resid 41 and name O) (resid 45 and name HN) 1.90 0.40 0.40

assign (resid 45 and name N) (resid 41 and name O) 2.85 0.55 0.55

!

assign (resid 42 and name O) (resid 46 and name HN) 1.90 0.40 0.40

assign (resid 46 and name N) (resid 42 and name O) 2.85 0.55 0.55

!

assign (resid 43 and name O) (resid 47 and name HN) 1.90 0.40 0.40

assign (resid 47 and name N) (resid 43 and name O) 2.85 0.55 0.55

!

assign (resid 44 and name O) (resid 48 and name HN) 1.90 0.40 0.40

assign (resid 48 and name N) (resid 44 and name O) 2.85 0.55 0.55

!

assign (resid 45 and name O) (resid 49 and name HN) 1.90 0.40 0.40

assign (resid 49 and name N) (resid 45 and name O) 2.85 0.55 0.55

(1) Mascioni, A.; Karim, C.; Barany, G.; Thomas, D. D.; Veglia, G. Structure and orientation of sarcolipin in lipid environments. *Biochemistry* **2002**, *41*, 475–482.
